# First Line Managers’ Experiences of Shaping Organisational and Relational Preconditions for Patient Safety in Swedish Intensive Care Units During the COVID‐19 Pandemic: A Qualitative Study

**DOI:** 10.1155/jonm/2665196

**Published:** 2026-08-29

**Authors:** Karin Berggren, Mirjam Ekstedt, Patrik Lynga, Peter Sackey, Lena Swedberg, Eva Joelsson-Alm, Anna Schandl

**Affiliations:** ^1^ Department of Perioperative and Intensive Care, Sodersjukhuset, Stockholm, Sweden; ^2^ Department of Clinical Science and Education, Sodersjukhuset, Karolinska Institutet, Stockholm, Sweden, ki.se; ^3^ Department of Health and Caring Sciences, Linnaeus University, Växjö, Sweden, lnu.se; ^4^ Department of Learning, Informatics, Management and Ethics, Karolinska Institutet, Stockholm, Sweden, ki.se; ^5^ Department of Cardiology, Sodersjukhuset, Stockholm, Sweden; ^6^ Department of Physiology and Pharmacology, Karolinska Institutet, Stockholm, Sweden, ki.se; ^7^ Department of Patient Safety and Quality, Sodertalje Hospital, Stockholm, Sweden

**Keywords:** crisis management, first-line managers, patient safety

## Abstract

**Objectives:**

The COVID‐19 pandemic placed extraordinary demands on intensive care units (ICUs), posing significant challenges to patient safety and underscoring the critical role of healthcare managers. Although leadership has been widely studied during the pandemic, little is known about how first‐line ICU managers experienced their work supporting staff and shaping organisational and relational preconditions for patient safety. This qualitative study aimed to address this gap by exploring first‐line ICU managers’ descriptions of their work during the pandemic.

**Methods:**

Individual interviews were conducted with 15 first‐line managers (nurses and physicians) who had operational responsibility for ICU staff, including nurses, physicians and assistant nurses, during the first wave of the COVID‐19 pandemic (1 March–30 September 2020). Participants were recruited from five ICUs across three Swedish hospitals. Data were analysed using thematic analysis.

**Results:**

The first‐line managers’ experiences were described in three themes: Balancing resources, workload and the well‐being of ICU staff; fostering a responsive and adaptive work environment; and building trust and collaboration. According to the first‐line managers, they experienced conflicts between maintaining efficiency and ensuring preconditions for patient safety, often struggling to balance care with limited resources. They explained how flexibility, continuous learning and mutual trust contributed to an adaptable healthcare environment that enabled the identification of safety risks.

**Conclusions:**

This study highlights the unique role of first‐line managers in shaping organisational preconditions for patient safety in ICUs during the COVID‐19 pandemic. Their leadership was enacted in real time, embedded in clinical care, shaped by ethical complexity, resource constraints and the need to support staff well‐being. The findings reveal how adaptive leadership, characterised by flexibility, continuous learning and trust, enabled managers to respond effectively to rapidly changing conditions and maintain patient safety under pressure.

**Implications for Nursing Management:**

The results underscore the importance of equipping first‐line managers with leadership competencies that promote adaptability, trust‐building, and continuous learning, especially in high‐pressure environments such as ICUs. By demonstrating how managers balanced staff well‐being, resource constraints, and preconditions for patient safety, this study offers practical strategies to strengthen resilience in healthcare teams. These insights can inform leadership development programmes, organisational policies and system‐level reforms aimed at enhancing patient safety and preparedness for future healthcare crises.

## 1. Introduction

The COVID‐19 pandemic placed an extraordinary strain on healthcare systems worldwide, particularly in intensive care units (ICUs) [1, 2], where the rapid influx of critically ill patients challenged the capacity to maintain safe, high‐quality care [2]. During the pandemic, ensuring patient safety became more critical than ever, as healthcare professionals faced unprecedented risks, resource shortages and rapidly evolving clinical treatment routines [3, 4]. In this context, patient safety understood as the prevention of harm to patients [5] encompasses not only the avoidance of adverse events and errors, but also the creation of resilient systems, safe care processes and supportive organisational conditions that mitigate risk and promote high‐quality care. It thus emerged as a major concern, requiring coordinated efforts across all levels of healthcare [6–8].

Patient safety is increasingly conceptualised as an emergent property of healthcare systems, shaped by organisational and work environment conditions rather than solely by individual performance or the absence of adverse events [9]. Research on patient safety culture and work environments has demonstrated that leadership support, teamwork, communication, psychological safety and staffing conditions are central to safe care delivery, particularly in high‐risk settings such as ICUs [10–12]. First‐line managers play a crucial role in shaping these conditions through their everyday managerial practices.

The importance of leadership in promoting organisational resilience has been highlighted in several contexts [13], and management attitudes and behaviours have been shown to play a decisive role in motivating staff to actively contribute to safety initiatives and uphold safe practices [13–16].

Within the healthcare system, first‐line managers play a key role in maintaining quality standards and ensuring patient safety [17]. Their responsibilities typically include direct supervision of clinical staff, implementing clinical practice guidelines, allocating resources and balancing organisational demands from upper management with the needs and conditions of their units, including staff and patients. Their roles are often described as being “in the middle,” managing both downwards (towards staff) and upwards (towards senior leadership), which requires a mix of task‐oriented, relational and change‐oriented leadership skills [18]. Despite the recognised importance of leadership in such high‐risk environments as the ICU, existing research has primarily focused on leadership strategies, staff resilience and the general experiences of healthcare workers [19–21]. Few studies have examined how managers themselves described their daily managerial work to support staff and sustain preconditions for safe care delivery during the COVID‐19 pandemic. Moreover, the limited qualitative research available has not sufficiently explored healthcare managers’ own perspectives during crises. Existing interview studies have primarily focused on the experiences of frontline staff [19, 22] or have explored leadership in broad terms, without specifically addressing how first‐line managers worked to maintain these conditions for safe care delivery during the pandemic [23–27]. This gap in the literature is particularly concerning given the complex and dynamic nature of ICU care [28], where leadership decisions can have immediate and profound implications for patient outcomes. The current study aimed to explore how first‐line ICU managers experienced their work in supporting staff and shaping organisational and relational preconditions for patient safety during the pandemic.

## 2. Methods

### 2.1. Study Design

A qualitative descriptive design was used, and data were collected through individual interviews using semistructured questions [29]. The methodology helps understand experiences in a social context [30] and effectively describes phenomena that represent informants’ responses [31]. The study is based on constructive epistemology [32], which suggests that reality is not singular or objective, but rather co‐constructed through individuals’ experiences, interpretations and interactions. From this perspective, knowledge is understood as emerging through individuals’ subjective processes of meaning‐making as they engage with and interpret their world [33]. The Swedish Ethical Review Authority approved the study (2021‐00324, with the amendment 2023‐02527‐02). Participation was voluntary, and all participants provided written informed consent before the interview. The study adhered to the COREQ checklist for qualitative research reporting [34].

### 2.2. Setting and Participants

In Stockholm, adult emergency care is provided at six emergency departments, while intensive care is offered at seven hospitals across the region. Intensive care is characterised by continuous monitoring and advanced treatments of patients [35]. The ICU is staffed by multidisciplinary teams whose professional competence often overlaps, enabling close collaboration in patient care [36]. Before the COVID‐19 pandemic, Stockholm had around 90 intensive care beds. When the pandemic began, ICU capacity was rapidly expanded to manage the sharp increase in critically ill patients. Although there were some local variations, most ICUs shared similar organisational structures and faced comparable challenges [37]. During the initial phase, ICU capacity was increased three‐ to fourfold, and staff transitioned to extended 12.5 h shifts. As in other European countries, nurse assistants, registered nurses, physiotherapists, and physicians from other departments and even nonmedical staff were recruited to help meet the surge [38]. All healthcare workers were required to wear full protective equipment at the bedside, including masks, gloves, visors, and plastic aprons.

First‐line nurse and physician managers with operational responsibility for ICU staff—including nurses, physicians and assistant nurses—during the first wave of the COVID‐19 pandemic (1 March to 30 September 2020) were eligible for inclusion. Participants were recruited from five ICUs across three hospitals in the Stockholm region of Sweden.

A purposive sampling technique based on profession and work experience was used to select participants for the study [39]. Eligible individuals were identified by the head nurses at each participating unit and were invited to participate through email from one of the researchers. The participants received both verbal and written information about the study’s purpose and rationale. One of the researchers then contacted the informants by email or telephone to provide additional information about the study and to schedule a suitable time for the interview.

### 2.3. Data Collection

Semistructured individual interviews were conducted between 5 September 2023 and 9 April 2024 by two researchers (Karin Berggren and Patrik Lynga). Ten face‐to‐face interviews were conducted in a room at the hospital, and five interviews were conducted via video meetings. The interviews were audio‐recorded and lasted between 18 and 56 min, with a mean time of 35 min. The researchers developed an interview guide to ensure coverage of various aspects. Each interview began with questions about age, workplace, and work experience. These questions were followed up with the question “*Can you share specific examples of how you ensured patient safety while leading your organisation during the COVID-19 pandemic?*” This question aimed to encourage the participants to share their stories and experiences. The full interview guide is attached in Supporting File 1. The interviews were transcribed verbatim by a professional transcriber and checked for accuracy by two researchers (Karin Berggren, Anna Schandl). The research team comprised six nurses (Karin Berggren, Eva Joelsson‐Alm, Mirjam Ekstedt, Patrik Lynga, Anna Schandl, Lena Swedberg) and one physician (Peter Sackey), all of whom are experienced researchers.

### 2.4. Data Analysis

The study employed Braun and Clark’s six‐phase thematic analysis approach, which offers flexibility and emphasises the researcher’s active role in identifying, interpreting and organising themes within qualitative data. This method is well‐suited to capturing the depth and complexity of participants’ narratives, allowing for a nuanced understanding of their experiences and perspectives [40]. The research team was engaged in a reflexive and iterative process to identify patterns of meaning across data [40]. Each researcher contributed their individual perspectives and engaged with the data from different viewpoints, which enriched the analysis. The analysis process began with two researchers (Karin Berggren, Anna Schandl) familiarising themselves with the interview transcripts. Initial coding was carried out by systematically identifying and labelling meaningful segments of the data. These codes were then organised into preliminary themes by grouping related codes into broad categories, capturing recurring patterns. The themes were reviewed by all researchers (Karin Berggren, Eva Joelsson‐Alm, Mirjam Ekstedt, Patrik Lynga, Anna Schandl, Lena Swedberg, Peter Sackey) and refined through several rounds of reflection and discussion to ensure they accurately represented the study’s aim. Each theme was defined and named to reflect the core meaning and relevance. A final thematic structure was developed, and illustrative quotations were selected to support and exemplify the findings.

### 2.5. Rigour

Throughout the study, methodological rigour and trustworthiness were ensured by adhering to the qualitative principles outlined by Lincoln and Guba [41]. Credibility was strengthened by selecting informants among nurse and physician managers working in intensive care during the COVID‐19 pandemic, which increased the likelihood of illuminating various aspects of patient safety. Dependability was supported by a detailed account of the methodology and ensured through a systematic and transparent analysis process. Confirmability was achieved through triangulation, which enabled the identification of multiple perspectives and nuances within the data. The researchers maintained a reflexive approach throughout the process by engaging in open dialogue and continuously reflecting on the research process [42]. The team’s varied academic and clinical expertise contributed to a deeper understanding and interpretation of the data. Transferability was enhanced by a contextual description of the participants, supported by demographic information and an overview of the context in which the study was conducted [41]. To enhance the credibility of the findings, participant quotations were used to anchor the analysis in the original data.

## 3. Findings

Fifteen first‐line ICU managers participated in the study, comprising 12 nurses and three physicians, most of whom had substantial managerial experience (Table 1).

**TABLE 1:** Descriptive information about the first‐line managers included in the study (*n* = 15).

	Nurses	Physicians
Total number	12	3
Sex (female)	9	1
Age in years, median (range)	55 (40–65)	50 (48–58)
Work experience in years, median (range)	8 (3–21)	7 (5–8)
Highest formal leadership education, n		
Master of Science (1 year)	2	—
Master of Science (2 years)	1	—
Leadership courses	9	3

The first‐line managers’ experiences were described in three themes: Balancing resources, workload and well‐being of ICU staff; fostering a responsive and adaptive work environment; and building trust and collaboration. The themes and subthemes are presented in Table 2.

**TABLE 2:** Overview of the themes and subthemes.

Themes	Subthemes
Balancing resources, workload, and the well‐being of ICU staff	•Conflicts between efficiency and safety
	•Caring for employees
	•Building effective teams

Fostering a responsive and adaptive work environment	•Protective leadership
	•Creative and flexible work procedures
	•Adaptive decision‐making

Building trust and collaboration	•Relying on employees’ judgment
	•Shared responsibility and collaboration

### 3.1. Balancing Resources, Workload and the Well‐Being of ICU Staff

#### 3.1.1. Conflicts Between Efficiency and Safety

Participants described how patient safety efforts during the pandemic involved managing the conflicts between available resources and the need to provide safe care. First‐line managers reported difficulties in delivering the requested treatments due to staff shortages. To address this, they explained that treatment protocols were adjusted, and larger treatment areas/rooms were established to enable care with fewer staff members. They described frequent discussions about how to prioritise patient treatments to manage workload without compromising patient safety. First‐line nurse managers stated that they regularly assessed the necessity of tasks such as basic hygiene care to reduce assignments considered less critical. They noted that the goal of providing equitable treatment guided care decisions, although assessment criteria were adapted depending on the number of patients. Several participants mentioned that the safety margins became smaller, and the limits of care were continuously adjusted to respond to the changing conditions to provide the best possible care under the prevailing conditions.

*“The level of care we provide is always associated with the amount of workload…”* First‐line manager 14.

#### 3.1.2. Caring for Employees

The first‐line managers described concerns about the increased safety risks when staffing levels were too low. They reported that efforts were made to provide reasonable working conditions to retain ICU staff and manage the high patient flow. The participants explained that they tried to ensure staff had access to practical necessities such as food, protective equipment and psychological support. They also highlighted the importance of providing clear and sufficient information about the disease and routines for personal protective equipment, which they said helped reduce uncertainty and stress among staff. The first‐line managers stated that their primary focus was on supporting the well‐being of employees, recognising that a cared‐for team was better able to maintain patient safety. By addressing the staff’s needs, they believed that conditions for safe care could be upheld during the crisis.

*“As first-line managers, we were facilitators for the staff, making sure there were enough people, food, and protective equipment. We focused more on the work environment and the staff than the patients.”* First‐line manager 4.

#### 3.1.3. Building Effective Teams

The first‐line managers described that forming and maintaining effective teams was important for delivering safe patient care. They explained that teams were assembled based on staff members’ skill levels to ensure appropriate competence and efficiency. Some first‐line managers reported making changes, such as reallocating staff or adjusting workloads when needed. They stated that having the necessary skills within the team helped support the delivery of high‐quality care. However, several participants noted that as the number of employees under their supervision increased, it became more difficult to maintain detailed insight into daily care activities. Despite these difficulties, they described continued efforts to ensure that the right combinations of skills were present to provide safe and effective care.

*“We tried to organise the staff based on their competencies. We learned how to combine individuals with varying skill levels to achieve a balanced and competent team.”* First‐line manager 10.

### 3.2. Fostering a Responsive and Adaptive Work Environment

#### 3.2.1. Protective Leadership

The first‐line managers described efforts to protect the employees from unnecessary disruptions, such as excessive administrative work or top‐down decisions they considered unrealistic. They explained that this approach was intended to help ICU staff remain focused on delivering safe and effective care to critically ill patients. Several first‐line managers reported being selective in the information they shared with their teams, aiming to pass on only relevant and necessary details. They also described acting as intermediaries by filtering out guidelines from hospital management and regional authorities so that only applicable and clinically meaningful directives reached their teams. This filtering was portrayed to support staff in maintaining safe practices. Participants observed that ICU staff were striving to uphold the usual standards of care, and they introduced simplified work procedures in response. These streamlined processes were described as helpful, particularly for newer team members with limited clinical experience, as they reduced complexity and supported safer task execution. However, some first‐line managers noted that simplified routines made it more difficult to maintain individualised and person‐centred care when patient needs became more complex. They also described challenges in determining which changes were acceptable, as deviations from standard safety practices were sometimes unavoidable due to a lack of alternatives. These situations required continuous assessment of risks and benefits to ensure that patient safety was preserved as far as possible.

*“I shielded them from unnecessary questions, decisions, or anything else I could take on to keep distractions away from the floor. There were all kinds of crazy suggestions, so I stood in front, like a wall, saying, ‘Just let us do the work.’”* First‐line manager 7.

#### 3.2.2. Creative and Flexible Work Procedures

The first‐line managers reported that the hospitals were inadequately prepared for the crisis, citing challenges such as insufficient medication storage, shortages of medical equipment and a lack of trained staff. They reported that persistent shortages required healthcare workers to find solutions outside established routines and guidelines. First‐line managers explained that they had to employ or develop new medical technologies, sometimes before full safety certifications were completed, as well as set up new facilities and create escalation plans. They described the need to develop solutions to emerging problems rapidly and to access accurate information on treatment strategies and staff protection quickly. To gain insights, some participants reported consulting international colleagues or using online resources such as Google.

Several first‐line managers stated that they were consistently available on site for ICU staff to consult. They described how staff could raise any concern—such as questions about medical reactions or equipment issues—and receive immediate feedback. This open communication was described as enabling quick and creative problem‐solving to reduce patient safety risks.

*“Safety was a top priority, but there was also plenty of room for creativity since we constantly had to find solutions. It wasn’t just about following routines, but about thinking: ‘What happens if we do this?’”* First‐line manager 6.

Some first‐line nurse managers reported difficulties in reaching and communicating with nurses as a group, primarily due to the lack of formal meetings. To overcome this, they described being present in the patient care area and engaging directly with staff. They noted that breaks and pauses provided opportunities for interaction. Participants described how the fragmented composition of the staff structure made it difficult to communicate and share information efficiently, and that meetings were sometimes held in the personal protective equipment donning area to receive prompt feedback on safety risks.

#### 3.2.3. Adaptive Decision‐Making

Participants described the management process as involving straightforward local decision‐making, without complex or time‐consuming administrative procedures. They reported feeling both responsible and authorised to make critical decisions instantly, which they viewed as essential for addressing safety‐related issues as they emerged. Several first‐line managers explained that decision‐making was often adaptive, responding to dynamic or unexpected situations as they occurred and in ways they perceived as necessary to maintain safe care delivery. They noted that the absence of clinically useful guidelines and policies, particularly for managing ethically difficult circumstances, such as the discontinuation of ICU treatment, prioritisation of care or allocation of equipment, added to their sense of responsibility. Some participants stated that clear directives were needed to support decision‐making and to communicate acceptable deviations from standard practices to maintain patient safety. First‐line managers described providing regular updates to keep staff informed about the evolving situation and available resources, emphasising that timely information was crucial for staff to perform their tasks safely and effectively. Participants also described using rapid decision loops—listening, acting and evaluating—which they said enabled continuous adjustments in response to emerging risks.

*“There was no hassle; we were the ones in charge, and you didn’t have to argue with procurement or take the long routes. We just went for it.”* First‐line manager 6.

Some first‐line managers emphasised the importance of maintaining operational control. At the same time, they expressed frustration with higher‐level management, describing how difficult decisions were often delegated to them without sufficient support, particularly during periods of critical resource shortages.

*“They were eager to push the actual responsibility further down the line. They pushed it to the absolute outermost branch, and I felt that it was not okay.”* First‐line manager 7.

### 3.3. Building Trust and Collaboration

#### 3.3.1. Relying on Employees’ Judgement

Participants explained that being physically present enabled them to gain a deeper understanding of the ICU team’s daily challenges and achievements, which in turn increased their awareness of safety risks and needs. Physical presence was described as an important way for first‐line managers to monitor care processes, identify emerging issues and support staff in maintaining safe practices. However, several first‐line managers reported that heavy administrative workloads occasionally limited their ability to be present on site.

In these situations, they described relying on a high level of trust in the competence and judgement of healthcare professionals to manage safety precautions. Informal, verbal reporting was described as a key mechanism for identifying safety risks and enabling timely adaptations to ensure that safe care could be maintained.

*“I don’t have a full grasp of it, and I must trust that the staff is competent enough to report any safety risks to me.”* First‐line manager 4.

#### 3.3.2. Shared Responsibility and Collaboration

In addition to supporting their teams and acknowledging the pressures faced by healthcare workers, first‐line managers described promoting a collectivistic approach. Participants emphasised the importance of shared responsibility and collaboration in meeting the growing demands of healthcare, noting that no individual was expected to manage the situation alone. Some first‐line managers reported that this collective mindset helped relieve pressure on individual staff members and was perceived as fostering teamwork and shared responsibility.

*“I did what I could to explain that we were in a state of emergency. The focus was on helping these people survive. We’re doing our best, and we can’t do any better right now.”* First‐line manager 11.

Participants explained that their initial efforts focused on securing the equipment needed for daily care. As the situation evolved, they described how key individuals and designated groups were appointed to take over responsibilities for materials, allowing first‐line managers to concentrate on personnel‐related issues, which they viewed as essential for sustaining safe care practices. Through collaboration across different operational areas and hospitals, several first‐line managers reported playing a central role in coordinating staff relocation. They described efforts to ensure that personnel were placed where needed, whether to reinforce existing ICUs or to support the opening of new units, a strategy to keep intensive care functioning effectively as conditions change.

*“We hired people who didn’t have healthcare education to help on the ward. For example, their tasks could include holding the hands of anxious patients or talking to them, and things like that. In this way, the ICU staff could focus on other, more important tasks with patients who were more ill, for instance.”* First‐line manager 12.

## 4. Discussion

This study reveals how shaping organisational preconditions for patient safety in ICUs was enacted through real‐time, relational and ethically complex decision‐making by first‐line managers. Their embedded role in both clinical care and team coordination enabled a unique form of adaptive leadership that prioritised trust, flexibility and continuous learning, offering new insights into how safety is maintained under extreme conditions.

The findings of this study align with principles of adaptive management in safety‐critical organisations, as outlined by Reiman et al. [43], emphasising flexible, learning‐oriented safety management in complex adaptive systems. In the ICU, such approaches are central to patient safety, as they enable timely decision‐making and coordination under conditions of uncertainty, high workload and rapidly changing clinical demands. Since safety depends on the system’s adaptive capacity [9, 13], the managers in our study strengthened this capacity by promoting transparent information, building trust and granting staff greater autonomy. These approaches enabled frontline teams to respond more flexibly to rapidly changing demands. These findings align with a recent systematic review identifying management strategies that support resilience in healthcare, including relationship‐building, navigating trade‐offs, enabling adaptation, fostering innovation, promoting contextual understanding, facilitating communication and encouraging collaborative learning [44]. Previous research has also shown that fostering trust and granting staff greater autonomy contribute to increased employee engagement and improved performance, thereby enhancing patient safety and quality of care [45, 46]. However, greater autonomy may also place increased responsibility on healthcare workers when difficult decisions must be made outside established routines, potentially creating a sense of personal risk‐taking. In our study, first‐line managers attempted to mitigate this burden by acting as a buffer against poorly adapted guidelines and top‐down decisions, thereby allowing healthcare workers to focus on direct patient care. Chemali et al. demonstrated that there is a risk that staff may feel isolated in their decision‐making and, in the worst case, be held accountable if something goes wrong [47]. Similarly, a mixed‐methods study by Escher et al. identifies “the fear of making a mistake” as the primary source of stress among ICU staff during the pandemic [48], underscoring the importance of the managerial actions that reduce pressure and support staff wellbeing. Without such support, stress, and deterioration of the work environment may negatively affect employee well‐being [49]. This emphasis on supporting staff aligns with broader findings in the literature, highlighting the importance of managerial support and healthy work environments. Braithwaite et al.’s systematic review similarly identifies positive relationships between workplace culture and patient outcomes [16].

Our results further highlight leadership qualities that supported these safety‐critical functions in practice. Presence, transparency, and empathy, also reported by Hayes et al. [50], were regarded as essential for maintaining situational awareness, trust, and communication. Reviews of nurse leaders’ experiences during the COVID‐19 pandemic likewise emphasised visible leadership, understanding of frontline conditions [51], and attention to staff wellbeing [52]. In our study, first‐line managers made substantial efforts to protect and support nurses and physicians, which may have contributed to patient safety by reducing fatigue‐related error risk and sustaining staff performance during periods of extreme demand. Protective leadership emerged as another important finding in our study, although this aspect was less emphasised in Hayes et al. [50].

In line with Nordin et al. [23], intensive care managers in our study described leading rapid and large‐scale transformations under conditions of uncertainty. They supported staff in prioritising care and coordinating newly assembled teams while balancing escalating demands with constrained resources. Although these efforts enabled continued care delivery, they also placed substantial strain on staff and organisational resilience. ICU managers described the pandemic response as a rapid and, at times, ad hoc reconfiguration of intensive care with immediate implications for patient safety. They experienced ongoing tension between rising demands and limited resources, requiring continuous prioritisation while striving to uphold patient safety standards, a pattern also identified in previous studies [2, 23, 53]. Participants further described how workforce shortages, knowledge gaps, ethical challenges, and material constraints combined to increase risk and stress in ICU care, consistent with research showing that similar constellations intensified pressure on healthcare teams during the pandemic [2, 23, 48]. Other research also demonstrates that adaptive actions differ across organisational levels: top‐level managers focus on strategic goals, trade‐offs, and long‐term organisational support [54], whereas first‐line managers and healthcare teams must continuously balance workload, individual risks, and local staffing demands in daily practice [55].

Pandemic‐related challenges revealed the necessity of adaptable work routines and highlighted the importance of preparation, effective leadership, and flexibility in future crisis responses [56]. Based on our findings, we recommend allocating resources to continuously and systematically collect and analyse information on safety risks during ongoing crises to support coordinated quality improvement efforts. The study also highlights the need for documents and organisational support structures to guide difficult decisions regarding care prioritisation and ethical issues, thereby reducing the moral burden placed on individual staff members. Future research should investigate models for real‐time data collection and analysis of patient safety risks to ensure that care remains safe and effective, even under extreme conditions.

### 4.1. Patient and Public Involvement

Although no patients were included as participants in this study, the findings were discussed with a group of patient representatives, most of whom had received ICU care for COVID‐19. Their reflections provided valuable insights that helped contextualise the first‐line managers’ experiences. Drawing on their encounters, they acknowledged the risk of mistakes under extreme working conditions and expressed concerns about the uncertainty surrounding the disease and the high‐risk care environment. Despite these challenges, both patients and their relatives reported overall satisfaction with the care they received. They particularly appreciated the staff’s efforts to communicate openly and share new information as it became available, which fostered a sense of safety and trust. These perspectives underscore the significance of leadership and communication in maintaining patient safety during crisis conditions.

### 4.2. Limitations

The study’s strengths lie in its multicentre design and interprofessional approach, which captured diverse perspectives from both nurse and physician first‐line managers across several ICUs. This diversity enhances the transferability of the findings and provides a richer understanding of how organisational and relational preconditions for patient safety were shaped during the COVID‐19 pandemic. The inclusion of different professional roles broadened the scope of experiences, although it also introduced interpretive challenges when compiling and analysing the data. A clear and transparent description of the research process, including details on data collection, thematic analysis and reflexive team discussions, supports the study’s trustworthiness [41]. However, the study has some limitations. Transcripts were not returned to participants for validation, and findings were not shared for feedback, which may affect confirmability. To mitigate this, the interviewers used clarifying questions during the interviews to ensure accurate interpretation. The study was geographically limited to the Stockholm area, which may impact transferability to other regions or healthcare systems. Nonetheless, the findings are likely relevant to similar ICU settings facing comparable organisational and pandemic‐related challenges.

## 5. Conclusions

This qualitative study highlights the unique role of first‐line nurse and physician managers in shaping organisational and relational preconditions for patient safety within ICUs during the COVID‐19 pandemic. The managers’ leadership was enacted in real time, embedded within clinical care and shaped by ethical complexity, resource constraints and upholding staff well‐being. The findings reveal that managing an enormous workload with limited resources required sensitive and adaptive leadership. Flexibility, continuous learning and trust were central to creating a responsive healthcare environment where safety risks could be identified and addressed. These insights contribute to the field of patient safety and nursing management by illustrating practical strategies for resilient healthcare leadership and informing future innovations in leadership development, particularly in high‐pressure and uncertain clinical contexts.

## Author Contributions

Karin Berggren: conceptualisation, data collection, formal analysis, writing–original draft, and writing–review and editing. Mirjam Ekstedt: conceptualisation, formal analysis, methodology, and writing–review and editing. Patrik Lyngå: data collection, formal analysis, and writing–review and editing. Peter Sackey: conceptualisation and writing–review and editing. Lena Swedberg: conceptualisation, formal analysis, and writing–review and editing. Eva Joelsson‐Alm: conceptualisation, formal analysis, and writing–review and editing. Anna Schandl: conceptualisation, validation, formal analysis, writing–original draft, writing–review and editing, and supervision.

## Funding

The study was supported by grants from the Swedish Patient Insurance Company (LÖF).

## Disclosure

The funders had no role in the design or conduct of the study.

## Conflicts of Interest

The authors declare no conflicts of interest.

## Supporting Information

Additional supporting information can be found online in the Supporting Information section.

## Supporting information


**Supporting Information** Supporting File S1. Description: Interview guide. The semistructured interview guide used for individual interviews with first‐line managers.

## Data Availability

The data that support the findings of this study are available from the corresponding author upon reasonable request.
