# Supplementary material for: First Line Managers’ Experiences of Shaping Organisational and Relational Preconditions for Patient Safety in Swedish Intensive Care Units During the COVID‐19 Pandemic: A Qualitative Study
Source: J Nurs Manag. 2026 Aug 29;2026:2665196. doi: 10.1155/jonm/2665196 (PMC13525475; doi:10.1155/jonm/2665196)
Supplement: Supplementary file 1 [file JONM-2026-2665196-s001.docx]

***Supplementary File S1. Interview guide.***

*The semi-structured interview guide used for individual interviews with first line managers.*

- From a leadership perspective, can you describe what you think is needed to maintain safe care?
- Can you tell me your experiences of patient safety when leading an organisation during the COVID-19 pandemic? Good experiences? Bad experiences? Challenges?
- How did you gather/collect information about the safety situation during the pandemic? How was the information used? How did you determine whether the risk level was acceptable or not?
- Today, can you describe any preparation your organisation has made to be prepared for a potential future pandemic?
- Is there anything more you would like to add?
